# Adrenal Tumors with Unexpected Outcome: A Review of the Literature

**DOI:** 10.1155/2015/710514

**Published:** 2015-03-25

**Authors:** Thomas M. Kerkhofs, Rudi M. Roumen, Thomas B. Demeyere, Antoine N. van der Linden, Harm R. Haak

**Affiliations:** ^1^Department of Internal Medicine, Máxima Medical Center, Ds. Th. Fliednerstraat 1, 5631 BM Eindhoven, The Netherlands; ^2^Department of Surgery, Máxima Medical Center, De Run 4600, 5504 DB Veldhoven, The Netherlands; ^3^Research School GROW, School for Oncology and Developmental Biology, Maastricht University Medical Centre, P.O. Box 616, 6200 MD Maastricht, The Netherlands; ^4^Department of Pathology, Stichting PAMM, Michelangelolaan 2, 5623 EJ Eindhoven, The Netherlands; ^5^Department of Radiology, Máxima Medical Center, De Run 4600, 5504 DB Veldhoven, The Netherlands; ^6^Department of Internal Medicine, Division of General Internal Medicine, Maastricht University Medical Centre, P.O. Box 5800, 6202 AZ Maastricht, The Netherlands; ^7^Department of Health Services Research and CAPHRI School for Public Health and Primary Care, Maastricht University Medical Centre, P.O. Box 616, 6200 MD Maastricht, The Netherlands

## Abstract

The finding of an adrenal mass should induce a diagnostic work-up aimed at assessing autonomous hormone production and differentiating between benign and (potentially) malignant lesions. The common differential diagnosis in adrenal incidentaloma consists of (non-)functioning adenoma, pheochromocytoma, myelolipoma, metastasis, and primary carcinoma. There remains a category of lesions that are hormonally inactive and display nonspecific imaging characteristics. We provide a succinct literature review regarding pathologies from this category. Imaging and histological characteristics are discussed, as well as clinical management. In conclusion, an adrenal mass may present a diagnostic challenge. After exclusion of most common diagnoses, it can be difficult to differentiate between possible pathologies based on preoperative diagnostic tests. Surgical resection of possibly harmful tumors is indicated, for example, lesions with malignant potential or risk of spontaneous hemorrhage. Resection of an obviously benign lesion is not necessary, unless problems due to tumor size are expected.

## 1. Introduction

Clinicians may be confronted with adrenal masses in four different scenarios. The first category comprises patients presenting with endocrinological symptoms suggesting adrenal origin, such as virilization or Cushing's syndrome as seen in selected adrenocortical adenomas and carcinomas. Hypertension, flushes, and headache may be signs of pheochromocytoma or aldosterone-producing adenoma (Conn's syndrome). Secondly, patients may present with nonspecific symptoms that turn out to be caused by an adrenal tumor such as pain, fatigue, weight loss, or the sensation of an abdominal mass. Thirdly, adrenal metastases might be found in the work-up of another malignancy, for example, lung cancer. Finally, an adrenal mass may be found incidentally during evaluation for nonrelated complaints: a so-called adrenal incidentaloma.

The common differential diagnosis includes six entities which account for the large majority of all adrenal masses. This will be discussed first. Secondly, we discuss a remaining category that consists of ten entities that are hormonally inactive and display nonspecific imaging characteristics.

## 2. Differential Diagnosis

The common differential diagnosis in adrenal incidentaloma consists of nonfunctioning adenoma, functioning adenoma, pheochromocytoma, and adrenocortical carcinoma. Myelolipomas and metastases from various malignancies are also common and should be included [1, 2]. The ranking by likelihood of these diagnoses varies depending on individual presentation. In general, most incidentalomas (70–80%) are benign adenomas which cause no symptoms. However, in 5–20% of patients who have no endocrine signs or symptoms, analysis reveals subclinical hypercortisolism [3–5]. Pheochromocytoma makes up about 1.5–14% of incidentalomas, adrenocortical carcinoma (ACC) is found in 1.2–11%, aldosterone-producing adenoma is found in 1.6–3.3%, and adrenal metastases are found in 1–18% [6, 7].

## 3. Diagnostic Work-Up

The diagnostic work-up should be aimed at assessing autonomous hormone production and differentiating between benign and (potentially) malignant lesions.

Evaluation of cortisol and (nor)metanephrine secretion should be performed in all patients presenting with an adrenal mass, even in absence of clinical signs of Cushing's syndrome or pheochromocytoma [3, 6, 58, 59]. Also, clinicians should be aware of the possibility of adrenal insufficiency in case of bilateral lesions. Screening for primary hyperaldosteronism by measuring plasma aldosterone concentration and plasma renin activity should be performed if hypertension and/or hypokalemia are present [6, 58]. The most accurate predictor to differentiate between benign and malignant masses is attenuation on unenhanced CT. If the lesion's attenuation value is ≤10 Hounsfield units (HU), malignancy is extremely unlikely [60]. In case of HU > 10, a contrast wash-out sequence should be performed. A wash-out > 50% after 15 minutes is indicative of adrenal adenoma. Combined use of attenuation measurement and washout values can be used to discriminate adenomas from other adrenal masses with 98% sensitivity and 92% specificity [61].

Percutaneous adrenal biopsy has high false negative rates and there is a risk of complications. Therefore, the only role of percutaneous biopsy in the evaluation of an adrenal mass is confirming metastatic disease in patients with known extra-adrenal malignancy and confirming the diagnosis of ACC when radical resection is deemed not possible [3, 58].

## 4. Remaining Pathologies: A Mixed Group

There remains a category of lesions that are hormonally inactive and display nonspecific imaging characteristics, which poses a diagnostic challenge. Here we discuss individual entities from this group. A summary of imaging and pathological characteristics of these lesions is provided in Table 1.

Primary adrenal lymphoma (PAL) is a rare finding with less than 200 cases described in the literature. In 70–80% of cases both adrenal glands are affected [8–11, 62]. On imaging studies, PAL typically presents as a large mass in which cystic or hemorrhagic components may be present. Homogeneous and heterogeneous lesions are reported in similar frequencies. Diffuse large-cell B cell lymphoma is the most commonly reported subtype, anaplastic large cell or T-cell lymphoma are only reported sporadically [8, 11–13]. Treatment consists of combination chemotherapy, sometimes preceded by surgery in cases of a large tumor mass [8, 14]. Prognosis depends heavily on treatment response, but a mean overall survival of 15 months has been reported [10, 14].

Liposarcomas account for 45% of all retroperitoneal soft tissue sarcomas. Five histological subtypes are known, of which well-differentiated liposarcomas (WDLS) and dedifferentiated liposarcomas (DDLS) are most commonly found retroperitoneally [16]. DDLS is found as a focal lesion with low attenuation on T1-weighed MRI within a well-delineated, lipogenic, and septated mass that is the WDLS in approximately 10% of all cases [17]. Histologically, the dedifferentiated area is characterized by atypical nonlipogenic stromal cells with hyperchromatic nuclei that are scattered in fibrous septa. With increasing grade of dedifferentiation, cellularity increases and nuclear atypia is more prominent. Despite often severe nuclear deformities, the mitotic rate is not very high [18]. Retroperitoneal liposarcomas are notorious for recurring and prognosis is poor: 5-year overall survival rates differ from 36 to 55% [19–21].

Schwannomas originate from Schwann cells in peripheral nerve sheaths. Approximately 3% of schwannomas are located in the retroperitoneal space, where it may involve the adrenal gland and/or mimic an adrenal mass [23, 24]. All schwannomas display benign behavior, except for a poorly defined proportion of the rare subtype melanotic schwannoma [25]. The appearance of a schwannoma on CT-scan is a round and well-circumscribed mass, hypo- or iso-intense compared to muscle that enhances after contrast administration [26]. On T1-weighted MRI images, signal intensity is intermediate and similar to muscle. On T2-weighted images, signal intensity is markedly increased [26]. Histologically, a schwannoma can be recognized by the presence of elongated spindle cells, organized in areas of both high and low cellularity, called Antoni A and B tissue [24, 26]. Immunohistochemical staining is positive for neuron-specific enolase (NSE), microfilament proteins, and S-100 protein, the neural protein in Schwann cells [24, 27].

To our knowledge, there are no reports on recurrent retroperitoneal schwannoma after radical resection.

Ganglioneuromas typically arise from primordial neural crest cells present in the adrenal medulla [28–30]. Calcifications may be apparent on CT-scan in 30%–60% of cases. Unenhanced attenuation values are relatively high: >25 HU. Biological behavior is benign in most cases, although malignant transformation is supposedly possible [28].

Idiopathic adrenal haematomas may be discovered as incidentaloma, due to abdominal complaints or due to adrenal insufficiency. Imaging characteristics vary from well-demarcated homogeneous masses to heterogeneous lesions suspect for periadrenal infiltration [33]. Adrenalectomy is often performed in order to obtain a diagnosis.

Adrenal cavernous haemangiomas are very rare and have only been described in individual case reports [36–39]. Recurrence after complete resection is not reported; however malignant transformation to angiosarcoma may be possible.

Adrenal angiomyolipomas are extremely rare with only five cases reported [40–44]. These tumors are classified in the family of perivascular epithelioid tumors (so called PEComas). It may be difficult to differentiate this tumor from (ad)renal carcinomas on imaging studies and even upon histological examination. The presence of both adipose tissue and cells positively staining for muscle and melanoma markers are required for definitive diagnosis.

Adrenal leiomyosarcomas and epithelioid angiosarcomas are also exceptionally rare. Concise histomorphological examination combined with positive staining of specific immunohistochemical markers is necessary to confirm the diagnosis [45, 50]. Invasion of periadrenal tissue and the occurrence of distant metastases are certainly possible, but complete resection in early stage could prevent this from happening [46].

Adrenal cysts form a subcategory which can be divided into pseudocysts, endothelial cysts, epithelial cysts, and parasitic cysts [52]. On CT imaging, differentiation from malignant cystic neoplasms or pseudocysts associated with malignant tumors is not possible [63]. Pseudocysts and endothelial cysts are both considered vascular lesions, the first originating from adrenal hemorrhage and the latter from a preexistent vascular or lymphatic malformation [53, 54]. Adrenal lymphangioma is a subtype of an endothelial cyst. Histologically, the diagnosis can be established by determining the endothelial origin of the cells through immunohistochemical staining (CD31, CD34, D2-40). Epithelial cysts are more difficult to characterize, as the adrenal gland lacks acini where such a cyst should originate from. An alternative explanation suggests embryonic origin, where the cyst would develop from displaced mesothelial tissue [53]. Parasitic cysts are very rare, mostly caused by infection with echinococcus. However, the adrenal glands are involved in less than <0.5% of infected patients [52]. Of note, all adrenal pathologies may display cystic degeneration which should not be confused with these four subtypes of adrenal cysts.

## 5. Conclusion

An adrenal mass may present a diagnostic challenge. If a diagnosis is not established after exclusion of the most common diagnoses, a category remains that consists of rare entities. It may be difficult or even impossible to differentiate between these pathologies based on preoperative diagnostic tests. Radical surgical resection is indicated in case of possibly harmful tumors, for example, lesions with malignant potential, risk of spontaneous hemorrhage, or increase in size over time. Clinicians should assess these issues using clinical judgment complemented with radiological evaluation of the lesion, aimed at characteristics summarized in the present study. This will result in resection of benign lesions, but this is inevitable given the uncertainty that may remain after complete diagnostic work-up. Surgical resection is not necessary if a lesion is judged to be certainly benign unless the size of the lesion causes problems, for example, due to a mass effect on other abdominal organs.

## Figures and Tables

**Table 1:** Summary of imaging and pathological characteristics of rare adrenal pathologies.

Diagnosis	Number of cases	Imaging characteristics		Pathological characteristics		Clinical behavior
		CT	MRI	Histology	Immunohistochemistry +	
Primary adrenal lymphoma [8–15]	<200	Mostly hypodense tumors, aspect homo- or heterogeneous, slight to moderate contrast enhancement.	Iso/hypointense in T1 and hyperintense in T2.	Atypical cells, anisokaryosis, hyperchromasia, necrosis.	Most common: CD45/CD20/CD40 (B-cell) CD3/CD30/CD43 (T-cell)	Malignant

Dedifferentiated liposarcoma [16–22]	>500	DDLS: nonlipogenic, heterogeneous node within a well-delineated, lipogenic, and septated mass that is the WDLS	WDLS: >75% fat, nonlipomatous components are prominent thick septa. Nodular nonadipose areas may be present. DDLS within WDLS: low to intermediate on T1 and intermediate to high on T2.	Atypical nonlipogenic stromal cells with hyperchromatic nuclei, scattered in fibrous septa. Cellularity and nuclear atypia increase with dedifferentiation. Mitotic rate typically <8/10HPF.	MDM2, CDK4	Malignant

Schwannoma [23–27]	>500	Round, well-circumscribed, hypo- or iso-intense compared to muscle, enhancement postcontrast.	Intermediate on T1 (isointense to muscle), mared increase on T2.	Elongated spindle cells in areas of both high (Antoni A) and low cellularity (Antoni B).	NSE, microfilament, S100	Benign

Ganglioneuroma [28–32]	>60	Att. > 25 HU, homogeneous aspect, calcifications in 30%–60%.	Hypointense on T1, heterogen. hyperintense on T2.	Ganglion cells, spindle cells, nerve fibres.	NSE, synaptophysin, S100, and CD57.	Benign Rare: transformation to malignant nerve sheath tumor.

Idiopathic adrenal haematoma [33–35]	>10	Variable: homo/heterogeneous depending on lesion's age.	High intensity on T1 in periphery of lesion suggests hemorrhage.	Hemorrhage, necrosis and hemosiderin.	—	Benign

Cavernous haemangioma [36–39]	>60	Heterogeneous, central cystic/necrotic components, calcifications, nodular peripheral enhancement postcontrast.	Homogeneous on T1, high intensity on T2.	Necrosis, cystic components, large vascular spaces, single lining of endothelium.	—	Benign Risk of spontaneous hemorrhage. Rare: transformation to angiosarcoma.

Angiomyolipoma [40–44]	<10	Heterogeneous: contains fat, possibly small enhancing foci.	—	Adipose tissue, smooth muscle fibres.	HMB45, MART1/MelanA, smooth muscle actin.	Benign Risk of spontaneous hemorrhage.

Epithelioid angiosarcoma [45–49]	>20	Irregular margins, nonhomogeneous density, calcifications.	High intensity on T2.	Vascular spaces lined by endothelial cells with epithelioid features, possibly pleomorphism.	Factor VIII, also CD34 and UEA-1 (less specific).	Malignant

Leiomyosarcoma [50, 51]	<20	Heterogeneous, possibly also liquid components.	—	Spindle-shaped neoplastic cells, nuclear pleomorphism, giant cell formation.	Smooth muscle actin.	Malignant

Cyst [52–57]	>600					
*Pseudocyst *	39%	Fibrous wall, no endothelial/epithelial lining, dependent on age septations, blood products, fluid-fluid level, or soft tissue component.	Intermediate/high density in T1, marked bright up in T2.	Usually unilocular, no endothelium. Contains brown/reddish fluid. Connective tissue walls calcificated/hyalinized.	—	Benign
*Endothelial *	45%	Thin wall (≤3.5 mm), smooth borders and pure cystic internal structure. Att. < 20 HU. No contrast enhancement.	—	Smooth endothelial lining, contains clear or milky fluid.	D2-40.	Benign
*Epithelial *	9%			Lined with cylindrical epithelium.	Calretinin and WT-1.	Benign
*Parasitic *	7%	Floating membrane or daughter cysts, septal or mural calcifications, coexistent hydatid cysts of other organs.	—	Thick, possibly calcificated walls, parasites within.	—	Benign

DDLS: dedifferentiated liposarcoma, WDLS: well-differentiated liposarcoma, Att: attenuation on unenhanced CT, HU: Hounsfield units, NSE: neuron-specific enolase, UEA-1: Ulex Europaeus Agglutinin 1, WT-1: Wilms tumor protein, and Immunohistochemistry +: Positive immunohistochemical staining.
